# Worsening Isolated Superior Mesenteric Artery Dissection on the Day After Discharge

**DOI:** 10.7759/cureus.38100

**Published:** 2023-04-25

**Authors:** Hideki Sasaki, Yoshiaki Sone, Yukihide Numata, Shinji Kamiya, Miki Asano

**Affiliations:** 1 Cardiovascular Surgery, Nagoya City University East Medical Center, Nagoya, JPN

**Keywords:** false lumen, collateral pathway, mesenteric ischemia, emergency department, isolated superior mesenteric artery dissection

## Abstract

A 59-year-old male was transported to the emergency department by ambulance with complaints of left-sided abdominal pain. Blood gas analysis revealed elevated lactate, and plain computed tomography revealed no bowel ischemic change. Contrast-enhanced computed tomography revealed isolated superior mesenteric artery dissection with mildly stenosed true lumen. The patient was treated with conservative management on admission. Staged fluid intake, oral prescriptions, and diet were commenced with attention to the symptoms. After four days of hospitalization, the patient was discharged with a stable condition. However, the patient returned to our hospital complaining of left lower back pain three hours after discharge. Contrast-enhanced computed tomography revealed an enlarged false lumen with a moderately stenosed true lumen. After a thorough discussion between vascular surgeons and interventional radiologists, conservative management was commenced on the second admission. The clinical course was uneventful, with proof of improved imaging findings.

## Introduction

Isolated mesenteric artery dissection (ISMAD) is rare, with an incidence of 0.06% [1]. Recently, literature concerning ISMAD, including etiology, diagnosis, classification, treatment, and outcomes, has been reported [2,3]. Although it is a life-threatening disease, the criteria and definitive treatment strategy are not clear. In addition, when the symptoms worsen and imaging studies show aggravation, the choice of treatment depends on the physician's discretion. In this report, we present a patient with ISMAD whose symptoms worsened on the day after discharge.

## Case presentation

**Figure 1:**
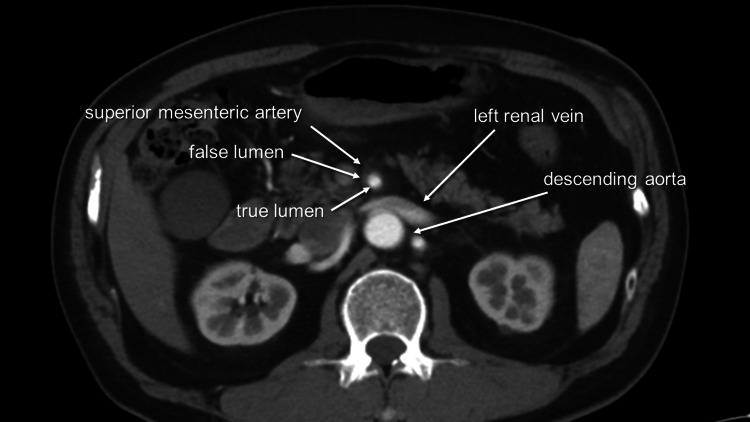
Axial view of superior mesenteric artery Mildly stenosed true lumen compressed by thrombosed false lumen.

**Figure 2:**
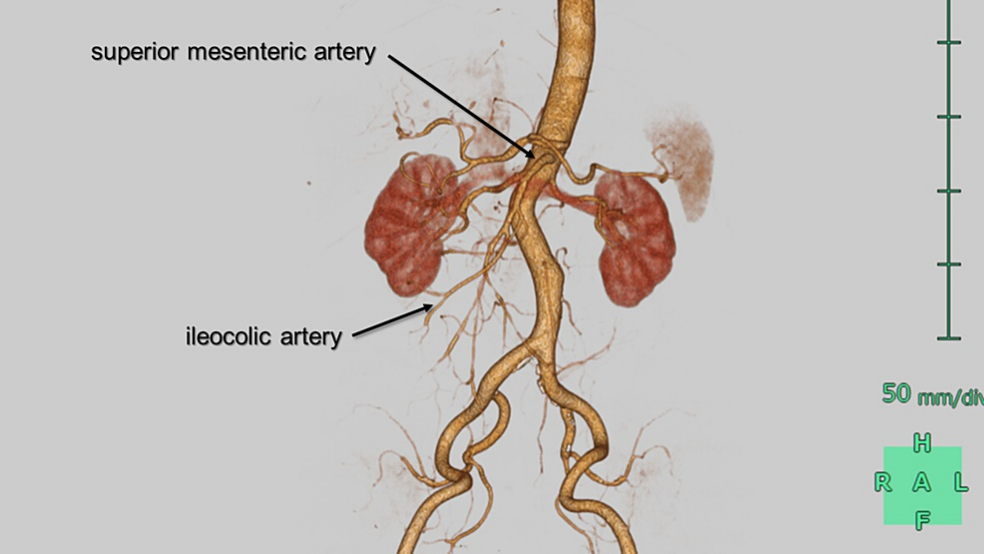
3D computed tomography of superior mesenteric artery Peripheral branches are weakly enhanced.

Upon arrival at the emergency department (ED), a 64-year-old male presented with sudden onset of severe left-sided abdominal pain that had persisted for an hour. His vital signs at the time of admission were notable for elevated blood pressure of 183/95 mmHg, a heart rate of 65 beats per minute, and hypothermia with a body temperature of 35.3 degrees Celsius. Physical examination revealed spontaneous pain in the abdomen, but no rebound tenderness was noted upon palpation. The patient had a past medical history of hypertension, dyslipidemia, and hyperuricemia. Blood gas analysis demonstrated respiratory alkalosis with a pH of 7.491, pCO2 of 33.7 mmHg, HCO3- of 25.5 mmol/L, base excess of 2.9 mmol/L, and elevated lactate levels of 3.5 mmol/L on arrival and 4.5 mmol/L an hour later. Plain computed tomography (CT) showed edematous changes around the superior mesenteric artery (SMA). Subsequent contrast-enhanced CT (CECT) imaging unveiled an isolated superior mesenteric artery dissection (ISMAD), in which the thrombosed false lumen caused a slight compression of the true lumen (Figure 1). Notably, despite the ISMAD, the peripheral arteries, including the ileocolic artery and its other branches, exhibited only weak enhancement (Figure 2).

**Figure 3:**
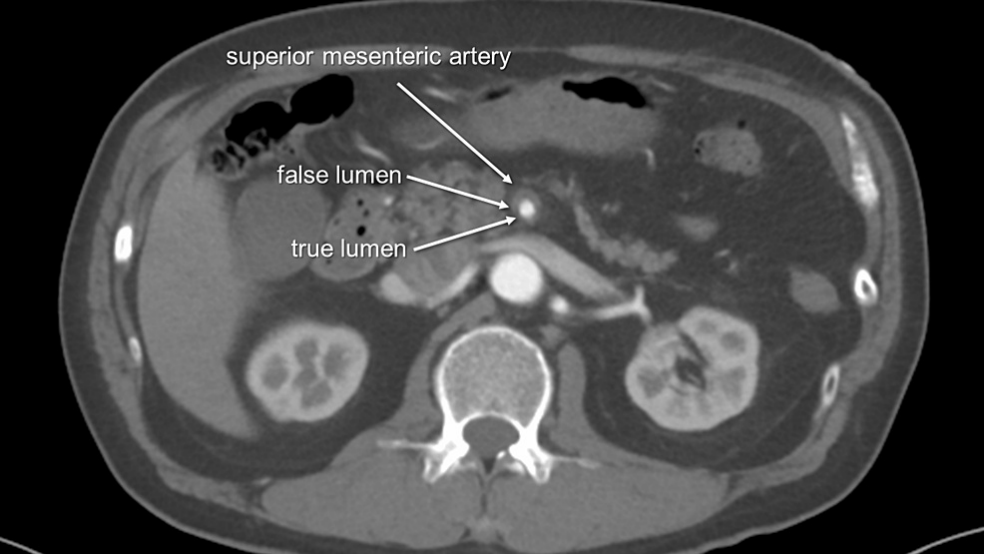
Axial view of superior mesenteric artery on the day after discharge Moderately stenosed true lumen compressed by a dilated false lumen.

The patient underwent a third blood gas analysis two and a half hours later, revealing a reduction in lactate levels to 2.8 mmol/L. The attending physician consulted with the vascular department, and the patient was admitted to our hospital for conservative management. Following a thorough discussion among vascular surgeons, the patient was prohibited from oral intake on the day of admission, and blood pressure was controlled via intravenous infusion of nicardipine hydrochloride. The following day, a plain CT scan revealed no evidence of intestinal edema, and the patient was permitted to drink water and prescribed oral medications including Losartan Potassium and Pravastatin Sodium. A diet was initiated two days after admission, and the patient reported no further symptoms. After four days of hospitalization, the patient was discharged. However, he returned to our hospital three hours later complaining of left-sided lower back pain. An emergent contrast-enhanced CT revealed worsening stenosis of the true lumen in the SMA with an enlarged false lumen (Figure 3). Although the true lumen was moderately to partially severely stenosed, collateral pathways enabled visualization of peripheral branches.

**Figure 4:**
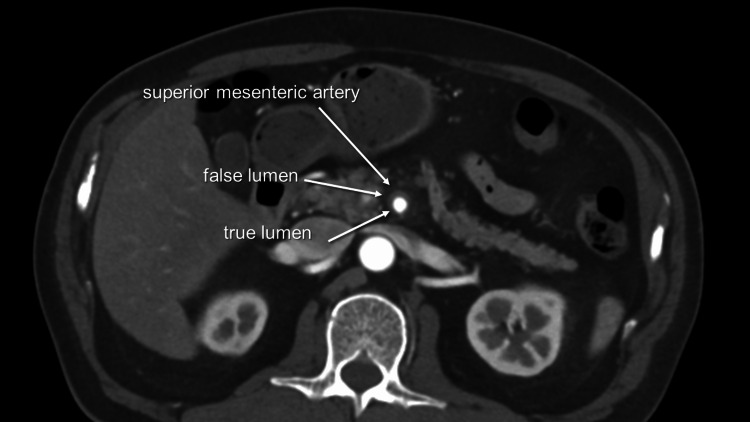
Axial view of superior mesenteric artery four days after the second admission Enlarged true lumen.

**Figure 5:**
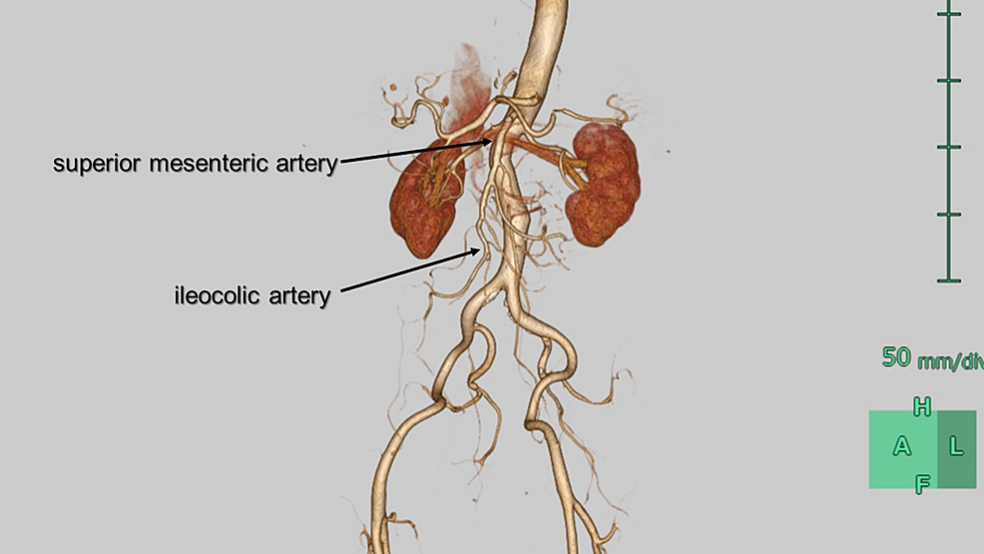
3D computed tomography of superior mesenteric artery four days after the second admission Better visualization of peripheral branches of superior mesenteric artery.

After a thorough discussion between vascular surgeons and interventional radiologists, we determined that conservative management, including strict blood pressure control and bowel rest, was the most appropriate choice. The patient was promptly readmitted to our hospital, and his symptoms were closely monitored throughout his stay. Upon conducting a plain CT scan two days after admission, we observed no edema of the intestines, allowing us to safely restart fluid intake and administer oral medications. Subsequently, the patient was able to resume a regular diet without any worsening of symptoms. Five days following readmission, we conducted a CECT, which revealed an enlarged true lumen (Figure 4). Three-dimensional CT revealed improved visualization of peripheral branches (Figure 5). As a result of his successful treatment and recovery, the patient was discharged home after a total of seven days of hospitalization without any complications.

## Discussion

ISMAD is a rare condition predominantly observed in males, particularly in the fifth decade of life [3]. Treatment can be chosen from three modalities: conservative, endovascular, and surgical management [2]. The current case sheds light on the difficulty of conservative management, which is associated with a lack of definitive criteria for selecting treatment. Although treatment consists of the aforementioned three modalities, conservative management is often used with acceptable outcomes [3]. Bowel rest is the first choice in conservative management, and antiplatelet, anticoagulation, and diet can be subsequently applied according to the patient's symptoms and serial CT findings. However, the duration of fasting, the timing of starting fluid intake, oral prescriptions, and diet are decided on a case-by-case basis because no definitive criteria exist. Although the true lumen was compressed by the thrombosed false lumen in the first CECT, the degree was not severe. During the first hospitalization, after one day of fasting, we started fluid intake, and subsequent oral prescriptions and diet were commenced, which did not worsen the patient's symptoms. However, considering that the false lumen suddenly enlarged its diameter on the day after discharge, it may have been better for the patient to continue hospitalization, which would allow for whole-body rest as well as bowel rest. Although it is difficult to explain inherent important factors, whole-body rest would play an important role in maintaining a stable condition. In addition, many factors, including blood pressure, the friability of the already dissected SMA, and the first intimal tear - especially its position and length - may have influenced the second attack leading to the aggravation of ISMAD. Although the diameter of the false lumen increased when the patient came back to the ED, the choice of treatment modality during the second admission is controversial. Endovascular treatment may be considered as a possible treatment option for severe true lumen stenosis if it exceeds 80% [4]. However, the decision to proceed with endovascular treatment would depend on various factors, including the location and severity of the stenosis, the patient's overall health, and their individual circumstances. Since the degree of stenosis was not severe on the CT scan conducted on the day after discharge, it may be appropriate to consider conservative management during the readmission. According to Sakamoto's classification, the current case falls under type IV, characterized by thrombosis of the false lumen, resulting in pressure on the true lumen [2]. There remains a possibility of recurrence in the medium to long term. While there are no established guidelines for determining whether endovascular or surgical intervention is warranted, the patient will be closely monitored using serial CT scans. It is also imperative to consider potential complications associated with invasive treatments.

When discussing the topic of organ ischemia, it is crucial to emphasize the importance of peripheral arterial circulation. Even in cases where the proximal segment of the superior mesenteric artery (SMA) is severely stenosed with thrombosed false lumen, the situation may not necessarily be problematic if collateral pathways from the inferior mesenteric artery, celiac artery, and other sources maintain peripheral circulation. However, if the dissection extends to distal branches such as the ileocolic artery, right colic artery, middle colic artery, and jejunal and ileal arteries, and peripheral circulation is compromised by thrombosed false lumen, intestinal ischemia can occur and potentially progress to necrosis. In such cases, surgical intervention, including bowel resection, may be necessary [5].

In summary, we encountered a patient with ISMAD who initially received conservative management, which unfortunately resulted in a worsening of symptoms on the day after discharge. The unpredictable nature of ISMAD recurrence and progression highlights the importance of individualized treatment approaches based on imaging studies and symptomatology.

## Conclusions

Although ISMAD is a rare clinical entity, it is crucial for healthcare providers to maintain a high degree of suspicion for its potential occurrence in clinical practice. Conservative management may be a valid option in cases where peripheral branches are visualized on CECT, even in the presence of a thrombosed false lumen compressing the true lumen.

When managing a patient with a recent onset of ISMAD who presents with recurrent symptoms, it is imperative for the physician to promptly obtain a CECT, carefully compare it to previous imaging studies, and select appropriate treatment modalities in collaboration with vascular surgeons and interventional radiologists. Given the current lack of definitive criteria or established treatment algorithms for ISMAD, a multidisciplinary approach is essential to carefully consider all potential factors and optimize patient outcomes.
